# The Surface Protein Fructose-1, 6 Bisphosphate Aldolase of *Klebsiella pneumoniae* Serotype K1: Role of Interaction with Neutrophils

**DOI:** 10.3390/pathogens9121009

**Published:** 2020-11-30

**Authors:** Chen-Hsiang Lee, Seng-Kee Chuah, Chia-Chi Chang, Fang-Ju Chen

**Affiliations:** 1 Division of Infectious Diseases, Department of Internal Medicine, Kaohsiung Chang Gung Memorial Hospital, College of Medicine, Chang Gung University, Kaohsiung 83304, Taiwan; mb90442043@hotmail.com (C.-C.C.); basophil2000@yahoo.com.tw (F.-J.C.); 2 Division of Gastroenterology, Department of Internal Medicine, Kaohsiung Chang Gung Memorial Hospital, College of Medicine, Chang Gung University, Kaohsiung 83304, Taiwan; chuahsk@adm.cgmh.org.tw

**Keywords:** innate immunity, virulence, pathogenesis, metastatic infection, protein expression

## Abstract

Hypermucoviscosity phenotypic *Klebsiella pneumoniae* (HV-*Kp*) serotype K1 is the predominant pathogen of a pyogenic liver abscess, an emerging infectious disease that often complicates septic metastatic syndrome in diabetic patients with poor sugar control. HV-*Kp*isolates were more resistant to neutrophil phagocytosis than non-HV-*Kp*isolates because of different pathogen-associated molecular patterns. The protein expression of HV-*Kp* after interaction with neutrophils is unclear. We studied KP-M1 (HV phenotype; serotype K_1_), DT-X (an acapsularmutant strain of KP-M1), and *E. coli* (ATCC 25922) with the model of *Kp-*infected neutrophils, using a comparative proteomic approach. One the identified protein, namely fructose-1, 6-bisphosphate aldolase (FBA), was found to be distributed in the KP-M1 after infecting neutrophils. Cell fractionation experiments showed that FBA is localized both to the cytoplasm and the outer membrane. Flow cytometry demonstrated that outer membrane-localized FBA was surface-accessible to FBA-specific antibody. The *fba* gene expression was enhanced in high glucose concentrations, which leads to increasing bacterial resistance to neutrophils phagocytosis and killing. The KP-M1 after FBA inhibitors and FBA-specific antibody treatment showed a significant reduction in bacterial resistance to neutrophils phagocytosis and killing, respectively, compared to KP-M1 without treatment. FBA is a highly conserved surface-exposed protein that is required for optimal interaction of HV-*Kp* to neutrophils.

## 1. Introduction

In the past 30 years, disseminated *Klebsiella pneumoniae* (KP) invasive syndrome has usually been observed in diabetic patients, especially in those with poor glycemic control [1]. In addition to causing primary liver abscesses with bacteremia, disseminated KP invasive syndrome causes metastatic endophthalmitis or meningitis [1]. This invasive syndrome has emerged in many countries, such as the US, Denmark and Taiwan [2,3,4].

Although the mechanism of invasive syndrome caused by KP in diabetics is still unclear, it is speculated that it may be related to vulnerable host and virulent strains. Diabetes itself can inhibit the chemotaxis of phagocytes, phagocytosis and bactericidal function [5], meaning that bacteria could infect diabetics. The capsule of KP has anti-phagocytic function against host phagocytes [5]. Furthermore, invitro studies revealed that the KP serotype K1 increased gene expression then increased synthesis of the capsular polysaccharide (CPS) in high glucose media, which is an important virulence factor for KP [6]. It was observed that phagocytosis of KP serotypes K1 and K2 by neutrophils was impaired in type 2 diabetic patients with poor glycemic control, and this was associated with an increased risk of metastatic complications [5,6]. However, 25% of patients with KP liver abscess do not have diabetes [2]; this reflects that the host factor may be one ofimportant factors rather than a decisive factor in the pathogenicity of disseminated KP invasive syndrome.

Previous studies on the pathogenic factors of KP strain have mostly focused on the study of genes related to capsule serotype and its control of capsule synthesis [7]. The main function of these genes and capsule is resistance to phagocytosis of phagocytes [8], which likely acts as a warrior’s shield rather than an attacking sword. The pathogenic factors might be closely related to bacterial surface proteins, but there has been no in-depth study yet. The research into protein expression level will thus be warranted.

In the innate immunity system, neutrophils play an important role in the fight against microbial infections. The neutrophils could migrate to the place where the pathogenic bacteria cause inflammation and execute phagocytosis of pathogens [9,10]. Our previous study revealedthat KP serotype K1 could extend the lifespan of neutrophils, making them suitable host cells for bacterial survival and multiplication after infection occurs [11]. The pathogens might be carried by neutrophils all over the body and form sporadic infections if the neutrophils cannot eliminate KP. Neutrophils may be used as “Trojan horses” for subsequent infection of other cells. The effect may contribute to the development of a distinctive invasive syndrome [11]. Although neutrophil immune responses are involved in disseminated KP infection, many questions remain unanswered. The investigation of the KP–neutrophils crosstalk can provide a comprehensive understanding of the pathogenesis of disseminated KP infection. Despite scattered efforts in this field, a systematic identification of interactions between host and bacterial proteins remains unavailable. The KP–neutrophils infection model was used to find out the special protein under the two-dimensional electrophoresis analysis of proteomics study. The aim of this study was to characterize the special protein on KP, its sub-cellular localization and its putative role in the pathogenesis of disseminated KP infection.

## 2. Materials and Methods

### 2.1. Ethics Statement

The Institutional Review Board of Chang Gung Memorial Hospital approved the protocol (permit No. 103-6901B). All subjects provided their written informed consent to participate in the study in accordance with the Declaration of Helsinki.

### 2.2. Bacterial Strains

A KP-M1 (ST23; serotype K_1_) strain, ahypermucoviscosity phenotypicisolate that was isolated from a patient with a liver abscess, an acapsular*K. pneumoniae* mutant of DT-X, a non-hypermucoviscosity phenotype strainwas isolated by subculture of strain DT-S (biotype *edwardsii*, capsule serotype K1, ST23) and *Escherichia coli* (ATCC 25922) were used as the representative strains in the following experiments. The lack of a capsule in DT-X was confirmed by staining with India ink [10]. Bacteria were routinely cultured at 37°C in Luria–Bertani (LB) medium. The hypermucoviscosity phenotype of the test isolates was determined using a modified string test [10].

### 2.3. Isolation of Neutrophils

Heparinized venous blood was obtained from five healthy adult volunteers. A standard density gradient separation method was performed to isolate human neutrophils from these healthy subjects’ whole blood using commercially available separation media—that is, a mixture of sodium metrizoate and Dextran 500. Pooled serum was obtained from these volunteers and stored in aliquots at −20°C until required.In the following studies, replicate tests were performed using neutrophils from different volunteers.

### 2.4. Opsonization and Infection of Neutrophils

KP-M1, DT-X, and *E. coli* were grown for 18 h in Trypticase soy broth (TSB, Difco, Lawrence, KS, USA), washed twice with a large volume of saline solution and adjusted to 1.5 × 10^7^ cells/mL in Ham′s medium. Bacteria were opsonized in the presence of 5% fresh pooled human serum in Ham’s medium in 30 min and were added to the neutrophil cultures in pooled serum at a multiplicity of infection of 1 bacteria/neutrophil under 37 °C [11].This test was repeated 5 times with neutrophils isolated from five separate volunteers.

### 2.5. Protein Extraction

Briefly, following 30 min of infection with KP-M1, DT-X, and *E. coli* (as described above), neutrophils (4.5 × 10^6^ cells) were collected after 6 h and then re-suspended in 50 µL of radioimmunoprecipitation assaybuffer(Cell Signaling Technology, Danvers, MA, USA) supplemented with a protease inhibitor at −80 °C overnight and centrifuged at 16,100× *g* for 20 min at 4 °C to obtain cell lysates. The supernatants were collected and the extracts were precipitated with a 2-D Clean Up kit (GE Healthcare, Chalfont St. Giles, UK) according to the manufacturer′s instructions.After discarding the cell debris, the supernatant protein concentrations were determined using the Bio-Rad Protein Assay Dye Reagent Concentrate (Hercules, CA, USA) [11].

### 2.6. Two-Dimensional Gel Electrophoresis and Image Analysis

All methods used are standard [12].The protein sample was made up to 100 µg in 250 µL with rehydration buffer (8 M urea, 2% (*w*/*v*) CHAPS, 0.5% (*v*/*v*) of pH 4.0–7.0 NL IPG buffer (GE Healthcare, Chicago, IL, USA)) containing 6.2 mg/mL DTT. Additionally, an extra paper soaked with 1% DTT was placed near the cathode during 1D IEF. The mixtures were applied to ImmobilineDryStrip gels (IPG strips; pH 4–7 NL, 13 cm; GE Healthcare). IEF was initiated at a low voltage (30 V, 12 h; 500 V, 1 h; 1000 V, 1 h), and then raised to 8000 V for 6 h. The focusing IPG strips were immediately equilibrated in SDS equilibration buffer (6 M urea, 50 mM Tris-HCl (pH 8.8), 30% glycerol (*v*/*v*), 2% SDS (*w*/*v*), 0.002% (*w*/*v*) bromophenol blue) containing 10 mg/mL DTT for 15 min, and thereafter in the SDS equilibration buffer containing 25 mg/mL IAA for 15 min. Following equilibration, proteins were separated by 12.5% SDS-PAGE, which was run until the bromophenol blue dye reached the end of the gel. Gels were fixed for 30 min in 40% ethanol and 10% acetic acid, and subjected to silver staining, and the reaction was stopped with 3.65 g EDTA in 250 mL ddH_2_O. Individual pairs of silver-stained SDS-PAGE gels in which proteins between post-infection with KP-M1, DT-X, *E. coli* and neutrophils alone had been resolved werescanned with LabScan (GE Healthcare), and images of the spots were automatically analyzed using Image Master 2D software (GE Healthcare). Only the protein spots that appeared and differed by at least 2-fold in paired (compared neutrophils alone with post-infection with KP-M1)2D gels were subjected to in-gel digestion forMALDI-TOF/TOF mass spectrometric analysis. The gel pieces were then dehydrated and subjected to trypsin digestion. Mass spectra were acquired as the sum of the ion signals generated by target irradiation with a mean of 300 laser pulses using the FlexAnalysis system (Bruker-Franzen Analytik, Bremen, Germany). Peptide fingerprints were selected in the mass range of 700 to 4000 Da and were analyzed using the Mascot software package (ver. 2.2.04). Protein identification required detection of unique peptides. Proteins with more than two spectral counts were selected for further analysis. The peptide mass data corresponding to each spot were searched against the SwissProt and NCBI database using Mascot (Matrix Science, Boston, MA, USA) search engines for peptide matching. The matched peptides that were considered as potent candidates had the highest Mascot score (≥65) and a peptide sequence coverage of 20 percent of the matched peptide.

### 2.7. Preparation of Recombinant Fructose-Bisphosphate Aldolase (FBA) Protein Andproduction of a Rabbit Antiserum against Purified Recombinant FBA

The T7 Cloning and Expression System (Lucigen) was used to prepare FBA. The *fba* gene was amplified from KPM1 using oligonucleotide primers fbaA1-F; (5′-GAAGGAGATATACATATGTCTAAAATTTTTGATTCGTA-3′) and fbaA1-R; (5′-GTGATGGTGGTGATGATGCAGAACGTCGATCGCGTTCAG-3′) [13]. The amplicon ligated into the pETite C-His vector and plasmid was used to transform *E. coli* BL21 (DE3) pLysS. Transformants were grown to log phase, induced with 1 mM IPTG for 4 h and harvested by centrifugation. We used Ni-NTA Fast Kit (Qiagen) and recombinant 6x histidine-tagged FBA then affinity-purified under native conditions for protein purification. New Zealand White female rabbits were immunized subcutaneously four times at 2 week intervals with 30 mg of rFBA protein emulsified in Freud’s complete (first immunization only) or incomplete adjuvant. After three injections, the animals were test bled, boosted once more and sacrificed 10 days later.

### 2.8. Sub-Cellular Localization of FBA

The sub-cellular localized of FBA was conducted as proposed by Tunio et al. [13] In brief, cells from 100 mL of overnight LB broth cultures were harvested at 13,000× *g* for 2 min and the pellet re-suspended in 1 mL of EB buffer (10 mM Tris-HCl pH7.5, 10 mM MgCl_2_, 25% sucrose), then washed twice in the same buffer. Finally, the pellet was re-suspended in EB buffer and incubated for 10 min on ice. The preparation was centrifuged at 13,000× *g* for 4 min; following re-suspension in 0.4 mL of ice cold water, the mixture was incubated on ice for a further 10 min, followed by centrifugation at 13,000× *g* for 2 min. The supernatant, containing periplasmic proteins, was removed and stored at −20 °C. The remaining cell pellet (spheroplasts) were re-suspended into 0.4 mL of Tris-HCl (pH 7.5) and sonicated using a SONICS^®^ VCX 750 for 10 cycles (each cycle consisting of a 10 s burst followed by a 10 s cooling period) to release the cytoplasmic contents. Non-disrupted cells were removed by centrifugation at 5000× *g* for 1 min. The upper clear supernatant was transferred to a fresh tube and centrifuged at 17,000× *g* for 30 min. The supernatant (representing the cytosolic fraction) was removed and stored at −20 °C. The remaining pellet was re-suspended in 0.4 mL of 10 mM Tris-HCl pH 7.5, 10 mM MgCl_2_, and 2% Triton X-100. The sample was incubated at 37 °C for 30 min and then centrifuged at 17,000× *g* for 30 min. The supernatant, enriched for cytoplasmic membrane proteins, was removed and stored at −20 °C. The pellet (enriched for outer membrane proteins) was re-suspended in 1 mL of 10 mM Tris-HCl pH 7.5, 10 mM MgCl_2_, and 1% Triton X-100, incubated at 37 °C for 30 min and then centrifuged at 17,000× *g* for 30 min. The final pellet was re-suspended in 0.2 mL of 10 mM Tris-HCl pH 7.5 and stored at −20 °C.

### 2.9. SDS-PAGE and Immunoblotting

Samples were re-suspended in a loading buffer (50 mM Tris–HCl pH 6.8, 2% SDS, 0.1% bromophenol blue, 10% glycerol, 100 mM dithiothreitol), and heated for 5 min at 90 °C. Denatured proteins (10 µg/sample) were separated on a 10% denaturing polyacrylamide gel by SDS-PAGE and transferred to PVDF membranes (0.45µm, Millipore, MA, USA). Membranes were blocked for 1 h at room temperature with a 5% (*w*/*v*) non-fat dry milk solution containing 10 mM Tris–HCl pH 7.5, 140 mM NaCl, and 0.1% Tween 20 (TBS–T) before incubation overnight at 4 °C with the primary antibody anti-FBA, diluted 1/5000 with 1% BSA in TBS–T. After washing, the membranes were incubated for 1 h with a peroxidase-labeled secondary antibody (Goat anti Rabbit-HRP, Jackson, MA, USA) diluted 1/10,000 with 2.5% non-fat milk in TBS–T, and the labeled proteins were detected using Immobilon^TM^ Western Chemiluminescent HRP Substrate (Millipore, Burlington, MA, USA) [11].

### 2.10. Quantitative Reverse-Transcription Polymerase Chain-Reaction (qRT-PCR) Assay

For transcriptional analyses of KP-M1 *fba* gene, KP-M1 was grown in LB broth alone or LB broth containing 0.1% glucose, 0.5% glucose, and 0.5% glycerol then incubated 6 h at 37 °C. The cultures were centrifuged for 10 min in a microcentrifuge at room temperature and the pellet was quickly resuspended in Trizol solution (Invitrogen, Carlsbad, CA, USA) to isolate total RNA. RNA RT-PCR experiments were carried out with the High Capacity cDNA Reverse Transcription Kits (Applied Biosystems) using random primers. Quantitative real-time PCR was performed in an Applied Biosystems 7500 Instrument using Fast SYBR Master Mix (Applied Biosystems) with primers (forward: 5′-AAAATCAGCCCGCGTTTCAC-3′, reverse: 5′-CGAAGTTCAGGCTGTTGTGC-3′) [14]. All data were analyzed using the comparative threshold cycle 2^−ΔΔCT^ method with 23S rRNA as the endogenous reference at least 3times.

### 2.11. Competition Inhibition Assays

The KP-M1 bacteria were cultured 6hat 37 °C on LB medium containing 0.5% glucose (with 5 μM, 25 μM or 100 μM of 2,3-dimercaptopropanesulfonate (DMPS, SIGMA, Duren, Germany #SI-D8016) or the metal-chelating compound dipicolinic acid (DPA, SIGMA, New Delhi, India #FL-02321) and control with PBS) [15]. The bacterial colonies were irradiated with UV light for bacterial inactivation, followed by sub-culture in plates to confirm sterility after overnight incubation. The purpose of inactive bacteria colonies is to keep a consistent MOI (10:1) for the phagocytosis assay. The irradiated cultures were resuspended in carbonate buffer containing 0.1% fluorescein isothiocyanate (FITC). The FITC-stained KP (FITC-KP) cells were counted using a bacterial cytometer and a fluorescence microscope. Phagocytosis was measured using a standard assay [16]. With the technique, FITC-unstained (adherent) and FITC-stained (intracellular) bacteria can be quantitated. The percentage of ingested bacteria was counted at 60 min. Briefly, 10 μL of FITC-KP (representing 5 × 10^8^ cfu/mL) was added to each 990 μL volume containing a mixture of 100 μLof a neutrophil suspension (representing 5 × 10^6^ cells/mL), 100 μL of pooled normal human serum (10% *v/v* for opsonization), and 790 μL of PBS. The final volume was 1.0 mL and the multiplicity of infection was 10:1. Each tube was incubated in a shaking water bath at 37 °C and transferred to an ice bath at a designated time point. A FACScan emitting an argon laser beam at 488 nm was used to detect FITC fluorescence. A total of 20,000 cells were processed using Cellquest version 1.0 software (Becton, Dickinson and Company, Franklin Lakes, NJ, USA). Green fluorescence (FL1-H) intensity data (collected using a logarithmic amplifier) were displayed as single histograms. By processing phagocytosis mixtures of unstained and FITC-stained bacteria, the boundary of positive and negative fluorescence was determined and the percentage of ingested bacteria was assessed.

### 2.12. Human Leukocyte Bactericidal Activity Assay after Block FBA of KP-M1 with Anti-FBA Antibody

Bactericidal activity was measured using a standard assay method [17]. The KP-M1 (1 × 10^7^ cfu/mL) samples were cultured in LB broth containing 0.1% or 0.5% glucose, respectively, then opsonized with pooled human 10% serum and added to human neutrophils either in the presence or absence of 0.1 mg anti-FBA antibody at a multiplicity of infection of 10:1 for 30 min. Post-infection, samples (50 μL) were withdrawn at 0, 1, 4, 6, and 24 h separately then diluted with 2.45 mL of H_2_O (pH 11.0, adjusted by NaOH) to lyse the neutrophils and disperse the bacteria sufficiently to permit evaluation by a colony assay [18]. All tests were performed in triplicate to ensure reproducibility.

### 2.13. Statistical Analyses

Data obtained in experiments which were expressed as the mean ± standard deviation, that included a control group and one experimental group, were analyzed using unpaired Student’s *t*-tests. Data obtained from studies containing multiple experimental groups were analyzed by one-way analysis of variance (ANOVA) followed by Tukey’s posthoc tests. All statistical analyses were two-sided, and values of *p* less than 0.05 were considered significant.

## 3. Result

### 3.1. Characterization of Protein Profiles of Neutrophils Infected by Bacteria

**Figure 1:**
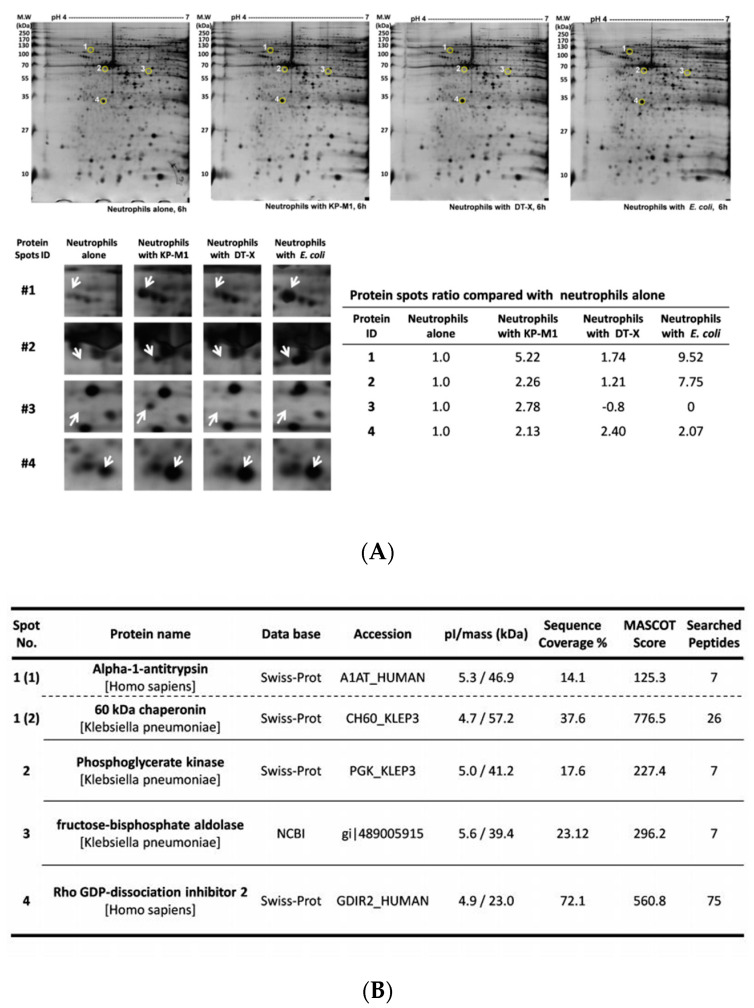
Proteome map of neutrophils infected by bacteria. Extracted proteins were separated by isoelectric focusing in the *p*I range of 4 to 7 in the first dimension and by 10% SDS-PAGE in the second dimension. Resolved proteins were visualized by sliver staining. Spot area indicated proteins with altered expression. Comparison of selected protein spot (arrow) extracted from neutrophils alone and neutrophils infected by different bacteria (**A**). The information of the expressed protein identified (**B**).

Following 30 min of infection with KP-M1, DT-X, and *E. coli*, neutrophils were collected 6 h post infectionand then were characterized to generate a proteome map. Protein samples were prepared, and over 200 protein spots were visualized. We initially performed 2-DE under a broad range of p*I* and molecular weight conditions with proteins extracted from infected neutrophils (data not shown). Several differences were observed and compared using ImageMaster software. These preliminary studies allowed us to select the optimal conditions to perform analysis at high resolution in the regions in which the main differences in the protein patterns were detected. Therefore, we focused on this p*I*range (pH of 4–7), as this also resulted in excellent spot resolution, and 4 spots were differentially expressed at least 2-fold greater in the infectedwith KP-M1 than in the neutrophils alone (Figure 1A). The comparative proteome analysis showed that neutrophils alters protein expression when infected by KP-M1, compared to infected by DT-X or *E. coli* ATCC 25,922 strain (Figure 1A). The unique protein spot (spot number 3) was identified as fructose-bisphosphate aldolase (FBA) using MALDI-TOF MS and the MASCOT search program (Figure 1B). The *fba* gene from KP-M1 was cloned and expressed in *E. coli*, which was identified bya rabbit antiserum against purified recombinant FBA under SDS-PAGE analysis (data not shown).

### 3.2. KP-M1 FBA Is Localized to Cytoplasm and Outer Membrane

**Figure 2:**
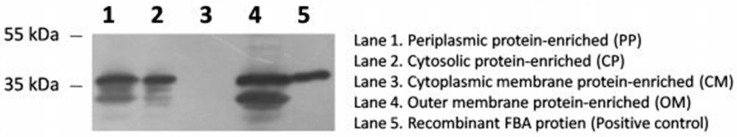
Sub-cellular localization of fructose-1, 6-bisphosphate aldolase (FBA).Periplasmic protein-enriched (PP, lane 1), cytosolic protein-enriched (CP, lane 2), cytoplasmic membrane protein-enriched (CM, lane 3) and outer membrane protein-enriched (OM, lane 4) fractions of KP-M1 and recombinant FBA (positive control, lane 5) were separated on a 10% acrylamide gel and probed in immunoblotting experiments. FBA was detected in PP, CP, and OM, but absent from CM.

The sub-cellular localized of FBA was investigated by sub-cellular fractionation followed by immunoblot analysis of the fractions. FBA was predominately detected in the outer membrane and cytosolic protein-enriched fractions but was absent from the cytoplasmic membrane-enriched fraction (Figure 2). FBA was also not detected in culture supernatants (data not shown). These findings demonstrate that KP-M1 FBA is a cytosolic protein that is found in the outer membrane.

### 3.3. Effect of Exogenous Glucose on KP-M1 fba Transcription

**Figure 3:**
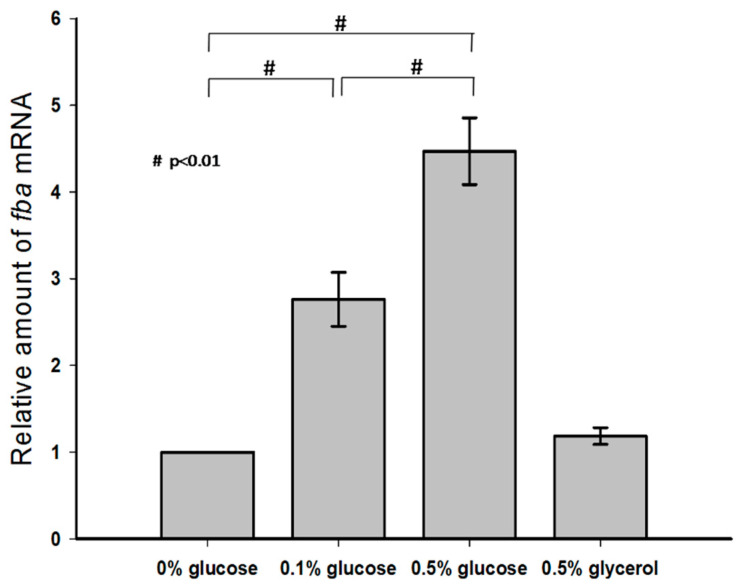
Exogenous glucose up-regulated *fba* transcription.Quantitative reverse-transcription polymerase-chain-reaction (qRT-PCR) assays were performed to investigate the expression of *fba* gene in KP-M1 in Luria–Bertani broth supplemented with glucose or glycerol as indicated at 37 °C 6 h agitation.

KP-M1 strains were cultured in LB broth alone or in LB broth supplemented with 0.1% glucose, 0.5% glucose, and 0.5% glycerol, and the mRNA levels of *fba* were measured by qRT-PCR. No statistical difference in the transcription of *fba* was observed between these growth conditions of LB broth alone and LB broth supplemented with 0.5% glycerol. However, growth in LB broth supplemented with 0.5% glucose increased the transcription of *fba* in a dose-dependent manner compared with growth in LB broth alone or in LB broth supplemented with 0.1% glucose (Figure 3).

### 3.4. Phagocytosis of KP-M1 after CompetitionInhibition Assays

**Figure 4:**
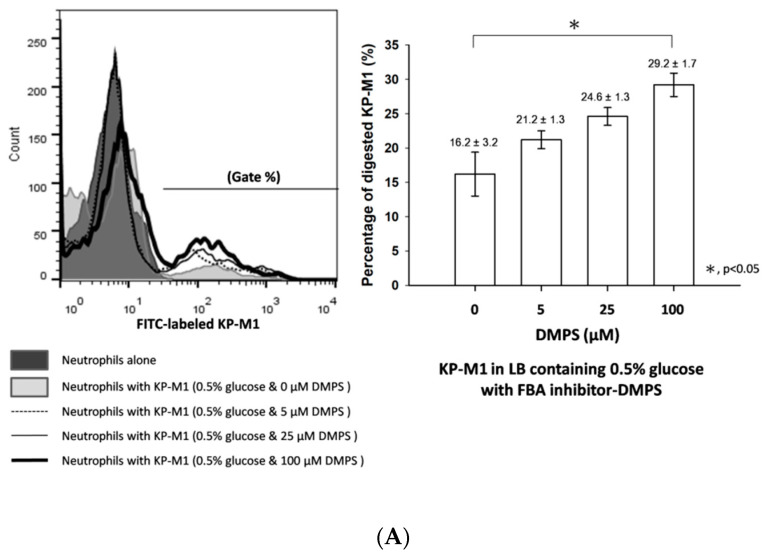

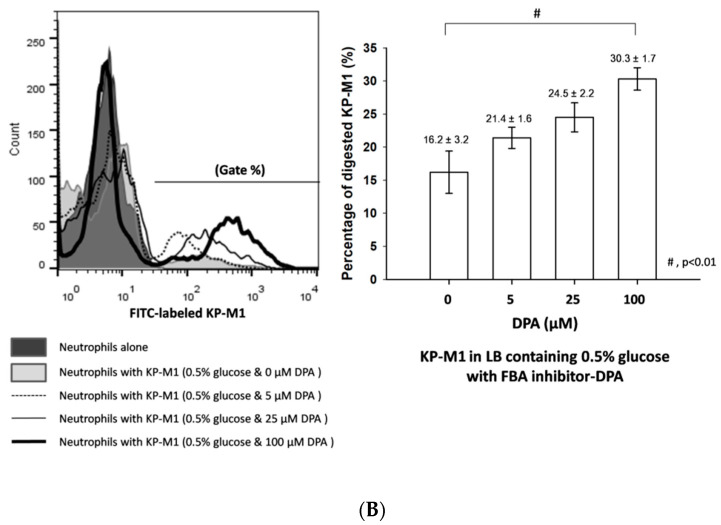
The effect of FBA inhibitors on KP-M1 against neutrophil phagocytosis. The KP-M1 bacteria were cultured 6 h at 37 °C on Luria–Bertani (LB) medium containing 0.5% glucose (with 5 μM, 25 μM or 100 μM of 2,3-dimercaptopropanesulfonate [DMPS] or the metal-chelating compound dipicolinic acid [DPA] and control with PBS). The KP-M1 strain on LB medium containing 0.5% glucose, compared to KP-M1 treated with different concentrations of DMPS (**A**) or DPA (**B**), was more resistant to neutrophil phagocytosis. The percentage of phagocytosis against KP-M1 increased significantly after treatment with 100 μM DMPS (*p* < 0.05, (**A**)) or 100 μM DPA (*p* < 0.01; (**B**)).

Metal-chelating inhibitors (DMPS or DPA) against FBAand FBA-specific antibody were not affected on its ability to grow invitro (data not show). The KP-M1 strain without FBA inhibitor treatment (compared to KP-M1 treated with different concentrations of DMPS or DPA) was more resistant to neutrophil phagocytosis (Figure 4A). The percentage of phagocytosis against KP-M1 increased in a dose-dependent manner after treatment with different concentrations of DMPS or DPA (Figure 4B). Compared with KP-M1 without FBA inhibitor treatment, the phagocytosis against KP-M1 increased significantly after treatment with 100 μM DMPS (*p* < 0.05) or 100 μM DPA (*p* < 0.01; Figure 4B).

### 3.5. Anti-FBA Antibody Enhances Human Neutrophils Bactericidal Activity against KP-M1

**Figure 5:**
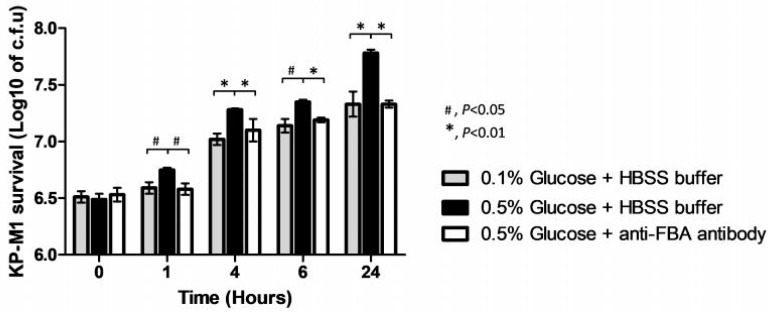
Anti-FBA antibody enhances human neutrophils bactericidal activity against KP-M1. The neutrophils bactericidal activity against KP-M1 in Luria–Bertani (LB) broth containing 0.5% glucose (black color bar) was significantly poor compared to those in LB broth containing 0.1% glucose (gray color bar). In the condition of LB broth containing 0.5% glucose (black color bar and white color bar), neutrophils’ bactericidal activity against KP-M1 was significantly improved when KP-M1 samples were treated with anti-FBA antibody (white color bar) compared to those without anti-FBA antibody treatment(black color bar) at 1, 4, 6, and 24 h post-infection.

After infection of neutrophils, we withdrew the samples at 0, 1, 4, 6, and 24 h post-infection to evaluate the colony counts. The neutrophils bactericidal activity against KP-M1 in LB broth containing 0.5% glucose was significantly poor compared to those in LB broth containing 0.1% glucose (Figure 5). In the condition of LB broth containing 0.5% glucose, neutrophils’ bactericidal activity against KP-M1 was significantly improved when KP-M1 samples were treated by anti-FBA antibody compared to those without anti-FBA antibody treatmentat 1, 4, 6, and 24 h post-infection (Figure 5). It showed anti-FBA antibody enhanced KP-M1 clearance by neutrophils.

## 4. Discussion

Our previous data suggest that KP serotype K1 can prolong the lifespan of infected neutrophils by delaying constitutive apoptosis within the first several hours of infection [11]. In the current study, theproteomics approach revealed a special protein, FBA expression of KP serotype K1 under KP-neutrophils infected mode. It was found both in the cytoplasm and the outer membrane of KP serotype K1 by cell fractionation experiments using immunoblot with rabbit anti-rFBA antibodies. FBA gene expression was enhanced in high glucose concentration, leading to increased bacterial resistance to neutrophils phagocytosis and killing. Finally, the FBA affects the KP serotype K1’sphagocytosis process.

Bacterial surface proteins might have several functions, including host’s cell adherence, specific receptors binding to modulate the immune response, nutrient acquisition and uptake of DNA from the environment [19]. In agreement with a previous study, FBA was largely distributed in KP NTUH-K2044 (K2044) strain, encapsulated with K1 antigen housekeeping pathways with significant over-representation and found mostly to be involved in translation and protein/carbohydrate metabolism [20]. To identify novel features of this protein, we performed gene regulation of FBA under environmentswith different glucose concentrations, and successfully constructed the *fba* gene, obtained recombinant protein FBA then prepared anti-FBA exclusive antibodies. Observing these experimental results, we found increasing transcription of *fba* in a dose-dependent manner in media with different glucose concentrations. Competition inhibition assays with FBA enzyme inhibitors increase the phagocytic effect of neutrophils against KP serotype K1. These resultsshow thatthe surface FBA expression of KP serotype K1 was increased under high concentration of glucose. The FBA inhibitors (DMPS and DPA) affect the protein expression and the anti-phagocytic capacity of the bacteria may be affected or destroyed, thereby effectively improving the phagocytosis of human neutrophils. The anti-FBA antibody study also showed elimination FBA of KP serotype K1 enhanced human neutrophils bactericidal ability. Our study recognized that FBA has additional functions beyond its housekeeping role in the central metabolism. To our knowledge, this is the first report demonstrating that a proportion of FBA is found on the cell surface of KP serotype K1. These results suggest FBA has a putative role in the pathogenesis of KP serotype K1 against neutrophils phagocytosis, which results in disseminated infection.

FBA is a central enzyme in the glycolysis and gluconeogenesis pathway that catalyzes the cleavage of its substrate fructose-1, 6-bisphosphate into glyceraldehyde 3-phosphate and dihydroxyacetone phosphate [21]. Different to other glycolytic enzymes [22,23], FBA has important functions other than its principal role as cytoplasmic enzyme in glycolysis. For instance, FBA seems to be crucial for the viability of either Gram-positive or Gram-negative bacteria, since its knockout makes many species unviable [24]. Thus, FBA is considered a “moonlighting” protein (protein with two or more dissimilar functions), and may have a significant role in both physiology and pathogenesis [25]. FBA has been found to localize to the surface of different pathogens [13,26,27]. It was found to mediate the adhesion to host cells [28] and was involved in modulation of the immune response [25]. Although we have shown that FBA is present on KP serotype K1 cell surface and is required for anti-phagocytosis against neutrophils, the role of FBA in this process is still unknown. It is possible that enzymatic activity of FBA plays an indirect role that is required for the suppression of FBA activity after KP serotype K1 treatment with a high dosage of FBA inhibitors, and then alters the effect of KP serotype K1 against neutrophils (Figure 4).

Furthermore, invitro studies revealed that, in high glucose media, KP serotype K1 increased gene expression and increased synthesis of the capsular polysaccharide, which is an important virulence factor for KP [6,29]. It was also observed that the phagocytosis of KP serotypes K1 and K2 by neutrophils was impaired in type 2 diabetic patients with poor glycemic control, and this was associated with an increased risk of metastatic complications [5,6]. In accordance with these data, our *fba* transcription result suggests that this protein is abundantly found in KP-M1 tested in a dose-dependent manner in high glucose media as well. Here, we show that gene *fba* is enhanced under defined conditions and appears to play a regulatory role in pathogenesis.

The function of FBA is crucial for bacteria, since its knockout makesmany species unviable [24]. One major limitation of our data is a lack of mutational analysis and functional complementation to identify additional functions of FBA in KP-M1. The CRISPR-Cas system can introduce multiple random off-target mutations in the genome [30]. It has been found to prevent the spread of plasmids and bacteriophages [31], and therefore limit the horizontal gene transfer by these mobile genetic elements. Only thosebacterialstrains that lack CRISPR-Cas can acquire other plasmids, while those strains that have CRISPR-Cas are protected from gaining these plasmids and thus maintain gene integrity. The KP-M1 isolate is a ST 23 strain and CRISPR-Cas cassette was detected by PCR [32] (data not shown), and thus mutation of gene *fba* is inaccessible in this study. Consequently, K1 serotype KP was the predominant strain leading to disseminated invasive KP syndrome, but only few KP serotype K1 isolates had resistance to multiple antimicrobials [1].

Taken together, these findings show that the immunoproteomics-based approach implemented in the present study identifies FBA in KP serotype K1 as a novel surface protein for the pathogenesis of disseminated KP infection, which warrants further studies.
